# Natural Plants Compounds as Modulators of Epithelial-to-Mesenchymal Transition

**DOI:** 10.3389/fphar.2019.00715

**Published:** 2019-07-30

**Authors:** Lorena Avila-Carrasco, Pedro Majano, José Antonio Sánchez-Toméro, Rafael Selgas, Manuel López-Cabrera, Abelardo Aguilera, Guadalupe González Mateo

**Affiliations:** ^1^Therapeutic and Pharmacology Department, Health and Human Science Research, Academic Unit of Human Medicine and Health Sciences, Autonomous University of Zacatecas, Zacatecas, Mexico; ^2^Molecular Biology Unit, Research Institute of University Hospital La Princesa (IP), Madrid, Spain; ^3^Department and Nephrology, Research Institute of University Hospital La Princesa (IP), Madrid, Spain; ^4^Research Institute of La Paz (IdiPAZ), University Hospital La Paz, Madrid, Spain; ^5^Renal research network REDINREN, Madrid, Spain; ^6^Molecular Biology Research Centre Severo Ochoa, Spanish Council for Scientific Research (CSIC), Madrid, Spain

**Keywords:** natural plants compounds, epithelial-to-mesenchymal transition (EMT), anti-fibrotic, anti-inflammatory, anti-oxidant agent

## Abstract

Epithelial-to-mesenchymal transition (EMT) is a self-regulated physiological process required for tissue repair that, in non-controled conditions may lead to fibrosis, angiogenesis, loss of normal organ function or cancer. Although several molecular pathways involved in EMT regulation have been described, this process does not have any specific treatment. This article introduces a systematic review of effective natural plant compounds and their extract that modulates the pathological EMT or its deleterious effects, through acting on different cellular signal transduction pathways both *in vivo* and *in vitro*. Thereby, cryptotanshinone, resveratrol, oxymatrine, ligustrazine, osthole, codonolactone, betanin, tannic acid, gentiopicroside, curcumin, genistein, paeoniflorin, gambogic acid and *Cinnamomum cassia* extracts inhibit EMT acting on transforming growth factor-β (TGF-β)/Smads signaling pathways. Gedunin, carnosol, celastrol, black rice anthocyanins, *Duchesnea indica*, cordycepin and *Celastrus orbiculatus* extract downregulate vimectin, fibronectin and N-cadherin. Sulforaphane, luteolin, celastrol, curcumin, arctigenin inhibit β-catenin signaling pathways. Salvianolic acid-A and plumbagin block oxidative stress, while honokiol, gallic acid, piperlongumine, brusatol and paeoniflorin inhibit EMT transcription factors such as SNAIL, TWIST and ZEB. Plectranthoic acid, resveratrol, genistein, baicalin, polyphyllin I, cairicoside E, luteolin, berberine, nimbolide, curcumin, withaferin-A, jatrophone, ginsenoside-Rb1, honokiol, parthenolide, phoyunnanin-E, epicatechin-3-gallate, gigantol, eupatolide, baicalin and baicalein and nitidine chloride inhibit EMT acting on other signaling pathways (SIRT1, p38 MAPK, NFAT1, SMAD, IL-6, STAT3, AQP5, notch 1, PI3K/Akt, Wnt/β-catenin, NF-κB, FAK/AKT, Hh). Despite the huge amount of preclinical data regarding EMT modulation by the natural compounds of plant, clinical translation is poor. Additionally, this review highlights some relevant examples of clinical trials using natural plant compounds to modulate EMT and its deleterious effects. Overall, this opens up new therapeutic alternatives in cancer, inflammatory and fibrosing diseases through the control of EMT process.

## Introduction

Epithelial-to-mesenchymal transition (EMT) is a tightly regulated physiological process implicated in tissue repair and in embryogenesis (Thiery et al., 2009). During EMT, epithelial cells undergo multiple morphologic, biochemical and genetic rearranges that gradually enable them to acquire a mesenchymal phenotype (Kalluri and Weinberg, 2009; Savagner, 2010; Kong et al., 2015).

We could consider two types of EMT, a physiological and a pathological EMT. The main characteristic of the physiological EMT is its ability to self-regulate, and it is related to embryonic development, organ formation, wound healing and tissue regeneration (Kalluri, 2009). In contrast, pathological EMT usually accompanies diseases and does not self-regulate. In this last case, EMT is an irreversible process and contributes to the failure of diseased organs (Thiery et al., 2009), which justifies the scientific investment into controlling such a process. Pathological EMT is present in many inflammatory and immunological diseases and leads to tissue fibrosis, angiogenesis, loss of organ function, cancer progression and metastasis (Corvol et al., 2009; Lee et al., 2006; Kalluri, 2009).

Although the involvement of EMT in some organ fibrosis, such as of the kidney, is controversial, more evidence about the role of pathological EMT has been reported in other organ fibrosis including that of the lung, the peritoneum and the heart, as well as in cancer progression (Yáñez-Mó et al., 2003; Rastaldi, 2006; Thiery et al., 2009; von Gise and Pu, 2012; Hertig et al., 2008).

EMT starts with the dissociation of intercellular junctions and the loss of microvilli and apical-basal polarity, followed bythe acquirement of a front to back polarity and an increased migratory capacity. In the latest stages of EMT, the cell increases its capacity to degrade the basement membrane and to invade the fibrotic compact zone. Cells that have undergone a mesenchymal conversion possess increased capacity to synthesize extracellular matrix (ECM) components as well as a large number of pro-inflammatory, fibrotic and angiogenic factors, including vascular endothelial growth factor (VEGF), inducing angiogenesis (Boutet et al., 2006; Yoo et al., 2006; Lopez-Cabrera, 2014; Grande et al., 2015; Lovisa et al., 2015; Cho et al., 2017; Kida et al., 2007).

To control the pathological EMT, many drugs and molecular measures with variable results have been tested, but this therapeutic target remains a challenge for current medicine (Aguilera et al., 2005). Targeting EMT can be a really interesting weapon for the treatment of many fibroproliferative, cardiovascular and autoimmune diseases, and other pathologies such as cancer.

### Molecular Mechanisms Involved in EMT Regulation

**Figure 1:**
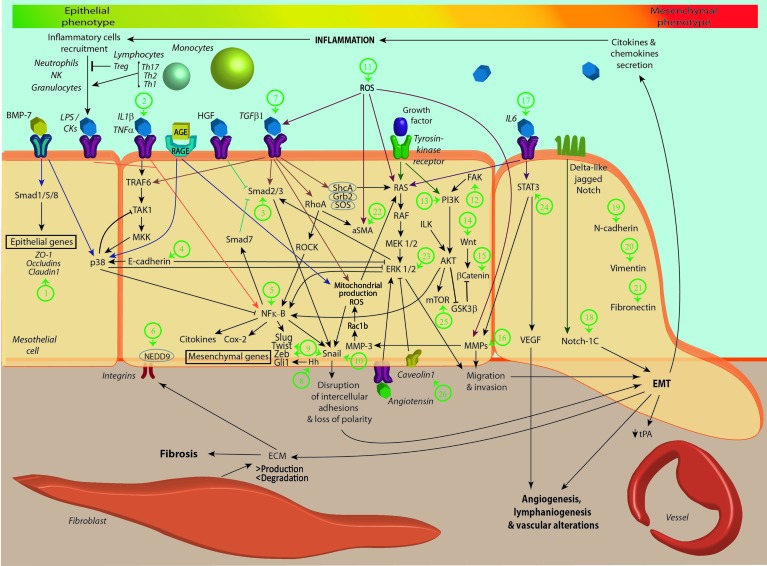
Key events during the development of epithelial-to-mesenchymal transition (EMT) and target pathways for therapeutic use of natural plant compounds (NPCs). The diagram shows key essential steps for the EMT course. Briefly, BMP7 and hepatocyte growth factor (HGF) are responsible for maintaining the cellular epithelial phenotype. Both molecules maintain E-cadherin and Smads1, 5, 7 and 8 expressions, and block Smads 2 and 3. On the other hand, inflammatory conditions triggered by exogenous or endogenous factors lead to a dysregulation of Th17/Treg equilibrium with IL6 synthesis that stimulates NFκB and induces EMT. Advanced glycation end products (AGEs) induce proteins structural crosslink, stimulate inflammatory response (monocytes), oxidative stress and finally uprregulation of Snail, leading to EMT. These effects are mediated through specific receivers called RAGES. Reactive oxigen species (ROS) generate oxidation and stimulate TGF-β, αSMA, RAS/RAF/MEK/ERK cascade and matrix metalloproteinases (MMPs) upregulation initiating cell migration. TGF-β initiates the classic route of EMT induction overexpressing Snail, Slug, N-Cadherin, Smads 2 and 3 and downregulating E-Cadherin and Smads 1, 5, 7 and 8. TGF-β also stimulates RhoA/ROCK and finally NFκB. Growth factors also stimulate the RAS/RAF/MEK/ERK cascade and MMPs upregulation, again inducing cell migration and invasion. Likewise they stimulate the P13k/ integrin-linked kinase (ILK)/ protein kinase B (Akt) cascade and GSK3β is blocked. Inflammatory cytokines such as IL6 also stimulates signal transducer and activator of transcription 3 (STAT3), vascular endothelial growth factor (VEGF) and MMPs inducing migration, invasion and angiogenesis. Finally Notch-1C directly induces mesenchymal genes such as N-Cadherin. The numbers in green indicate where each NPC acts. Many of them have an effect on several molecules or pathways. Depending on the target route over which the corresponding NPC acts, we have defined the following groups (green numbers). Group 1 acts by increasing ZO-1 expression: (Plumbagin), Group 2 acts by decreasing IL-1β action: (Baicalin), Group 3 acts by blocking Smad 2/3 phosphorilation; Paeoniflorin, Eupatolide, Gallic acid, Cairicoside E. Group 4 acts by up-regulating E-cadherin expression: α-solanine, Osthole, coumarin, Betanin, *Cinnamomum cassia* extracts, Genisteín, Withaferin A, Gedunin, *Celastrus orbiculatus* extract, Celastrol, Black rice anthocyanins, Duchesnea extracts, Cordycepin, Nitidine chloride, Phoyunnanin E, Epicatechin-3-gallate, Honokiol, Gallic acid, Piperlongumine, Brusatol, Berberine. Group 5 acts by inhibiting NFk-β signaling: Honokiol, Parthenolide, Baicalin and baicalein Group 6 acts by inhibiting NEDD9/Rac1 signaling: Acid plectranthoic. Group 7 acts by blocking TGF-β-1 signaling: Cryptotanshinone, Resveratrol, Oximatrina, Ligustrazina, Osthole, coumarin, Codonolactone, Betanin, Tannic acid, *Cinnamomum cassia* extracts Cairicoside E, Gentiopicroside, Genistein, Paeoniflorin, Gambogic acid, Arctigenin, Curcumin, Baicalin and baicalein, Baicalin and Cairicoside-E. Group 8 acts by downregulating hedgehog (Hn) signaling: Resveratrol, Sedum sarmentosum Bunge and extract Nitidine chloride Group 9 acts by decreasing Twist and ZEB expression: Resveratrol, Paeoniflorin, Jatrophone, Gedunin, Nitidine chloride, Plumbagin, Honokiol, Phoyunnanin E, Gallic acid, Piperlongumine, Brusatol, Nimbolide, Baicalin and baicalein. Group 10 acts by downregulation Snail expression: Resveratrol, Osthole, coumarin, Paeoniflorin, Gedunin, Celastrol, Nitidine chloride, Plumbagin, Phoyunnanin E, Piperlongumine, Berberine and Nimbolide. Group 11 acts by Inhibit Nrf2-mediated oxidative stress signaling pathway: Betanin, Salvianolic-acid-A and Plumbagin. Group 12 acts by Suppressing focal adhesion kinase (FAK)/AKT signaling: Phoyunnanin, Epicatechin-3-gallate, Gigantol and Eupatolide. Group 13 acts by blocking P13K/Akt cascade: Berberine, Nimbolide, and Curcumin. Group 14 acts by blocking Wnt/β-catenin Wnt signaling: Withaferin A, Jatrophone, Ginsenoside-Rb1 and Withaferin-A. Group 15 acts by down-regulated β-Catenin expression: Celastrol, Sulforaphane, Arctigenin, Plumbagin, Curcumin and Luteolin. Group 16 acts by down-regulating MMPs expression: α-solanine, Resveratrol, *Cinnamomum cassia* extracts, Paeoniflorin and *Celastrus orbiculatus* extract. Group 17 acts by inhibiting IL-6 activity: Baicalin and Polyphyllin-I. Group 18 acts by blocking Notch-1 signaling: Gedunin, Nimboliden and Luteolin. Group 19 acts by down-regulating N-cadherin expression: Tannic acid, Paeoniflorin, Gedunin, *Celastrus orbiculatus* extract, Celastrol *Duchesnea indica*, Cordycepin, Nitidine chloride, Honokiol, Phoyunnanin E, Gigantol, Gallic acid, Berberine and Nimbolide. Group 20 acts by down-regulating Vimentin expression: α-solanine Tannic acid, *Cinnamomum cassia* extracts, Paeoniflorin, Withaferin A, Jatrophone, Gedunin, *Celastrus orbiculatus* extract, Celastrol Black rice anthocyanins, *Duchesnea indica*, Nitidine chloride, Plumbagin, Phoyunnanin E, Gigantol, Gallic acid, Berberine and Nimbolide. Group 21 acts by down-regulation Fibronectin expression: Tannic acid, Cinnamomum cassia extracts, Jatrophone, Withaferin A, Black rice anthocyanins, *Duchesnea indica*, Epicatechin-3-gallate, Gallic acid and Berberine. Group 22 acts by down-regulating α-SMA expression: Betanin, Celastrol and Salvianolic-acid-A. Group 23 acts by blocking extracellular signal-regulated kinase (ERK) signaling: Arctigenin, Gigantol, Eupatolide and Nimbolide. Group 24 acts by blocking STAT- 3 signaling: Honokiol and Polyphyllin-I. Group 25 acts by blocking mammalian target of rapamycin (mTOR) signaling: Nimbolide. Group 26 acts by suppressing the cav-1 phosphorylation, stabilizating β-catenin: curcumin.

Mechanistically, EMT is a complex, dynamic and progressive process that affects the cellular architecture and requires a deep molecular reprogramming with new biochemical instructions (Lopez-Cabrera, 2014). The EMT process results from an integration of diverse signals transduction pathways and multiple triggering factors, including inflammatory cytokines, advanced glycation end products (AGEs), oxidative stress and hypoxia, transforming growth factor-β1 (TGF-β1)/Smads pathway, tyrosine kinase receptors, delta-like jagged Notch, caveolin (cav)-1, angiotensin receptor and integrins (**Figure 1**). A master molecule in the EMT induction appears to be TGF-β, nontheless the number of molecules and routes implicated in EMT is still growing (Lopez-Cabrera, 2014). The binding of the TGF-β1 to its “primary receptor” (receptor type II) permits the recruitment, trans-phosphorylation, and activation of the “signaling receptor” (receptor type I), also known as activin receptor-like kinase 5 (ALK5). Then, ALK5 is able to exert its activity to phosphorylate Smad2 and Smad3 (Masszi and Kapus, 2011). These receptor-activated Smads (R-Smads) form heterodimers with Smad4, a common mediator of the Smad pathways. These resulting Smad heterocomplexes are translocated into the nucleus, where they bind directly to DNA to regulate the transcription of target genes (Lopez-Cabrera, 2014). Another group of Smads, known as “inhibitory Smads” (e.g. Smad7), control TGF-β1-induced Smad signaling by preventing the phosphorylation and/or nuclear translocation of R-Smads and inducing receptor heterocomplex degradation (Lopez-Cabrera, 2014).

Among the R-Smads, Smad-3 appears to be the key mediator in TGF-β-induced fibrosis and EMT (Zhou et al., 2010). In this context, it has been shown that the inhibition of Smad3 activation and nuclear translocation blocks EMT (Zhou et al., 2010) and tissue fibrosis (Sato et al., 2003). Translocated Smad-3 into the nucleus controls TGF-β-responsive genes encoding integrin-linked kinase (ILK) (Massagué, 2000). The activation of ILK by β1-integrins lead to protein kinase B (Akt) and glycogen synthase kinase-3 (GSK-3)-beta phosphorylation (Massagué and Wotton, 2000).

Phosphorylated-Akt activates nuclear factor-κB (NF-κB) (Tan et al., 2002), which induces the expression of Smad-7 (Bitzer et al., 2000) emphasizing the self-regulated nature of the whole EMT process (**Figure 1**). On the other hand, phosphorylated-GSK-3β is inactive, what subsequently stabilizes β-catenin, released from the adherens junction, and activator protein-1 (AP-1) (D’Amico et al., 2000). When stabilized, β-catenin per se may induce EMT (Kim et al., 2002a), while AP-1 activates matrix metalloproteinase (MMP)-9 expression inducing the invasion of the ECM (Troussard et al., 2000).

The Smad-dependent pathways are not the only ways by which TGF-β1 regulate the EMT process. Smad-independent pathways also participate in TGF-β1-induced EMT. These pathways can either potentiate or modulate the outcome of TGF-β1-induced Smad signaling (Lopez-Cabrera, 2014). Amain Smad-independent signalling pathway activated by TGF-β1/receptor I interaction is the Ras homolog gene family member A (RhoA)/rho-associated, coiled-coil-containing protein kinase 1 (ROCK1) pathway. This route regulates cytoskeleton remodelling and cellular migration and invasion. RhoA also induces the expression of alpha smooth muscle actin (α-SMA) in a ROCK-independent manner (Masszi et al., 2003).

TGF-β1 also activates the H-Ras/Raf/extracellular signal-regulated kinase (ERK) pathway, necessary for the induction of transcription factor SNAIL1 expression and of EMT (Peinado et al., 2003; Barberà et al., 2004; Huber et al., 2004), cooperating with fibroblast growth factor (FGF), a potent inducer of the mentioned route (Peinado et al., 2003). SNAIL1 regulates EMT by inhibiting E-cadherin (Cano et al., 2000; Poser et al., 2001) and by inducing growth arrest and survival, which confer advantage to migrating transdifferentiated cells (Vega et al., 2004).

The Notch signalling pathway is another EMT-activating route, able to induce SNAIL1 and SNAIL2 expression, down-regulating E-cadherin. The TGFβ/Smads classical pathway is able to cooperate with different signaling routes. The association of the tumor necrosis factor (TNF)-receptor associated factor 6 (TRAF6) with the TGFβ receptor heterocomplex activates TGFβ-activated kinase 1 (TAK1) and, as a result, p38 and c-Jun N-terminal kinase (JNK) (Thiery et al., 2009). Other stimuli such as advanced glycation endproducts AGEs are able to induce EMT by acting on specific cellular receptors (RAGE) (Chen et al., 2016b). Reactive oxygen species (ROS) can also directly activate TGF-β, the production of ECM, MMP and RAS (Thiery et al., 2009) (**Figure 1**). Finally, the activation of mammalian target of rapamycin (mTOR) induces inflammatory processes mediated by T helper 17 (Th17) cells, TH17, which in turn also triggers EMT (Liappas et al., 2015).

### Active Substances Derived from Plants Capable of Regulating EMT

Natural plant compounds (NPCs) have been used for many years as a source of therapeutic substances and a structural basis for drug elaboration. Unique architectures that can lead to novel therapeutic agents are provided by nature (Newman and Cragg, 2007; Song et al., 2014; Khan and Gurav, 2017).

NPCs are bioactive elements isolated from natural sources (plants) that can regulate the EMT through anti-inflammatory, anti-fibrotic or antioxidant mechanisms (Boldbaatar et al., 2017). Bioactive natural components are presented in this review which delves into their mechanisms of action against EMT (**Table 1**).

**Table 1:** Natural plants compounds able to modulate epithelial-to-mesenchymal transition (EMT).

Target	Scientific name of plant	Active compounds	EMT-related signaling pathways	Type of study	References
*7,15,23*	*Asteraceae* plants	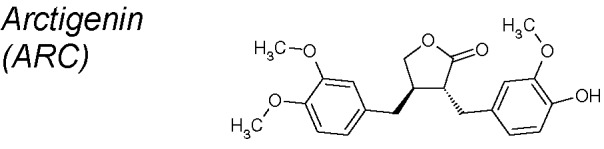	Represses TGF-β-induced phosphorylation of ERK and transcriptional activity of β-catenin	*In vitro*	Xu et al., 2017
*2,7,5,9*,*17*	*Scutellaria baicalensis* Georgi	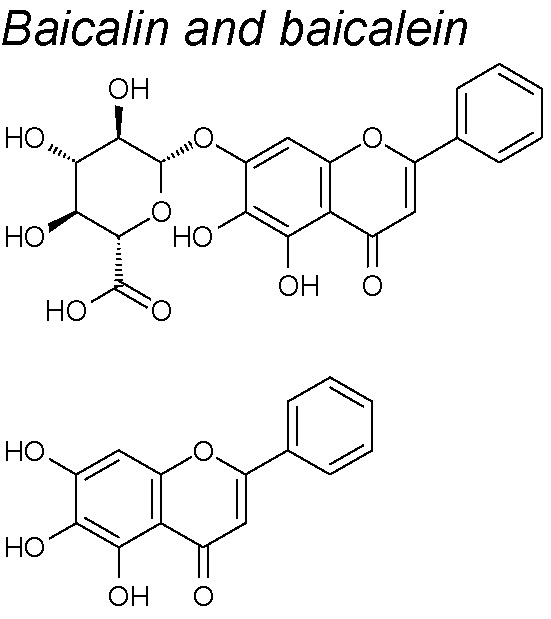	Blocks TNF-α and IL-1B, reduce TGF-β1, TNF-α, IL-6 and increase IL-10 (anti-inflammatory cytokine). Downregulates Slug expression and block NF-κB pathway signaling.	*In vitro*	Chung et al., 2015; Zheng et al., 2016
*4,10,13,19,20,21*	*Berberis vulgaris,aristata* *and aquifolium*	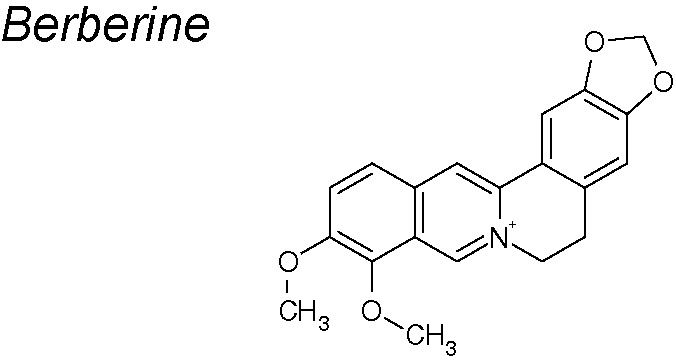	Increases E-cadherin and decreases N-cadherin, vimentin, fibronectin and β-catenin. Inhibits snail1, slug, and ZEB1. Blocks PI3K/AKT and RARα/RARβ.	*In vitro*	Kou et al., 2016
*4,7,11,22*	*Opuntia elatior* Mill.	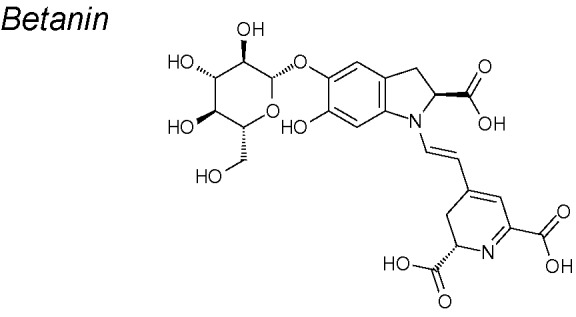	Blocks TGF-β signal pathway and modulates mRNA and protein expression of TGF-β, type IV collagen, α-SMA and E-cadherin and regulates oxidative stress and TGF-β pathway	*In vivo*	Sutariya and Brijesh, 2017
*4,20,21*	*Oryza sativa* L.	Black rice anthocyanins (BRACs). 9 anthocyanins have been detected in black rice (Hao et al., 2015)	Upregulates E-cadherin, and decreases fibronectin and vimentin expression	*In vivo* and *in vitro*	Sehitoglu et al., 2014; Hou, 2003; Zhou et al., 2017
*4,9*	*Bruceae fructus*	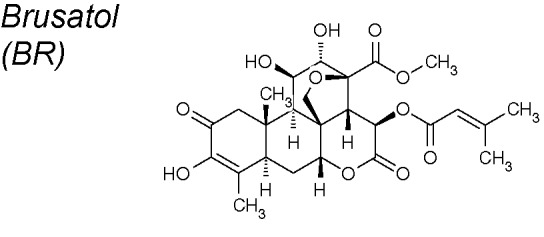	Increases E-cadherin mRNA expression and decreases Twist expression	*In vitro*	Lu et al., 2017
*2,7*	*Rosemary* (*Rosmarinus officinalis* L.)	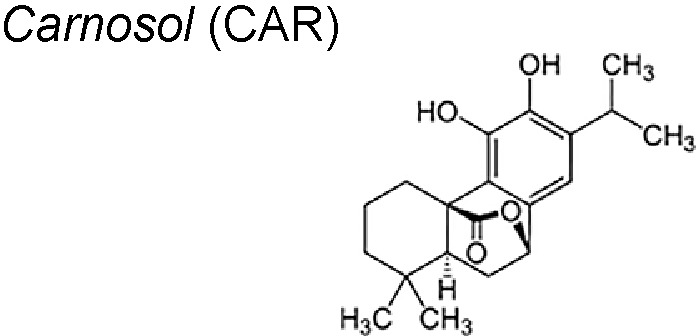	Controls the TNF-α/TGF-β-induced EMT and modulating the activation of miR-200c.	*In vitro*	Giacomelli et al., 2017
*4,10,15,19,20,22*	*Tripterygium wilfordii*	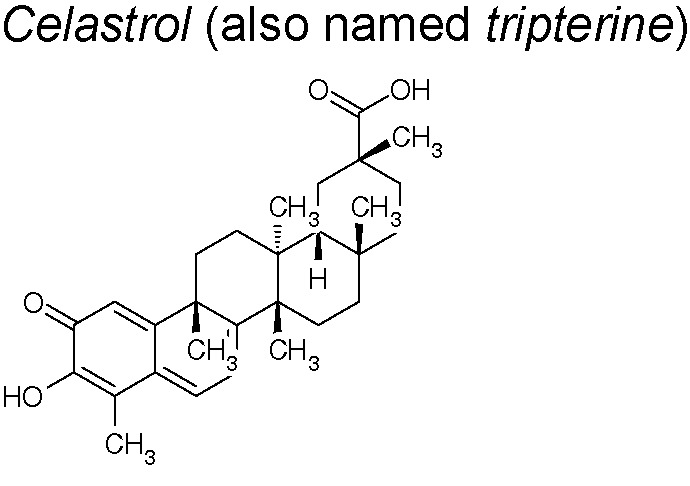	Upregulates E-cadherin and down-regulates N-cadherin, Vimentin and Snail	*In vivo* and *in vitro*	Lin et al., 2015
			Downregulates β-catenin, N-cadherin, vimentin, α-SMA, FSP-1 and collagen expression and inhibits heat shock protein 90 signaling	*In vivo* and *in vitro*	Divya et al., 2018
*3,7*	*Ipomoea cairica*	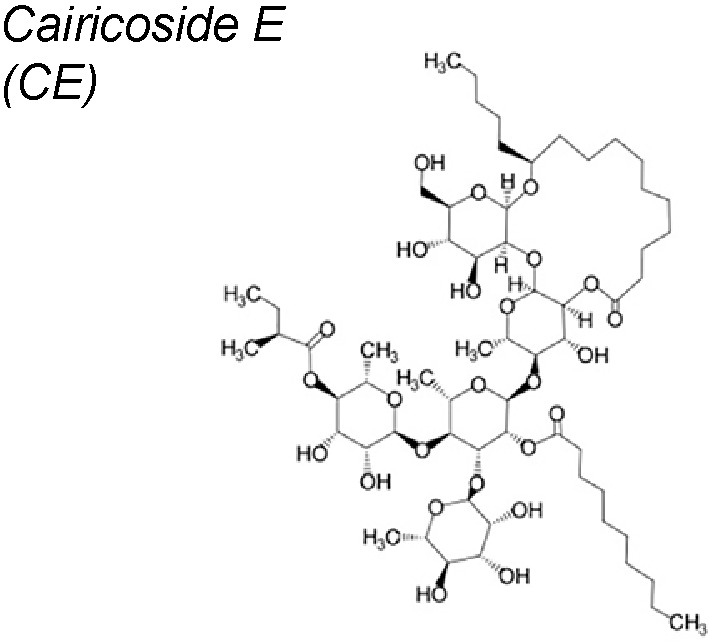	Down-regulates AQP5 expression and suppresses p-Smad2/3 induced by TGF-β1	*In vitro and in vivo*	Chen et al., 2017a
*4,16,19,20*	*Celastrus orbiculatus*	*Celastrus orbiculatus extract (COE)* There are 11 compounds in the stems (Li et al., 2012a).	Reduces angiogenesis by targeting the VEGF protein	*In vitro and in vivo*	Qian et al., 2012
			Activates MAPK and inhibits Akt signaling pathways	*In vitro*	Zhang et al., 2012a
			Inhibits Cofilin 1 signaling pathway, N-cadherin, vimentin, MMP-2 and MMP-9 protein expression and upregulates E-cadherin protein expression	*in vitro*	Wang et al., 2017b
*4,7,16,20,21*	*Cinnamomum cassia*	*Cinnamomum cassia extracts (CCE)* There are 15 compounds in the bark (Zhao et al., 2013)	Inhibits TGF-β1 by repressing MMP-2 and urokinase-type plasminogen activator also downregulating expression of vimentin and fibronectin and upregulating E-cadherin	*In vitro*	Lin et al., 2017
7	Atractylodes lancea	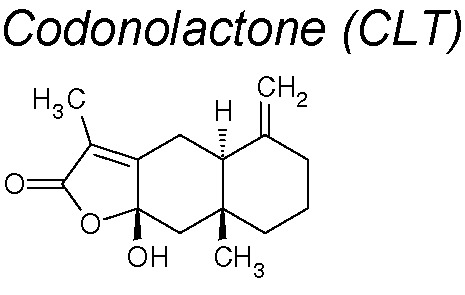	Suppresses of TGF-β signal pathway and Runx2 phosphorylation	*In vivo* and *in vitro*	Fu et al., 2016
*4,19*	*Cordyceps sinensis*	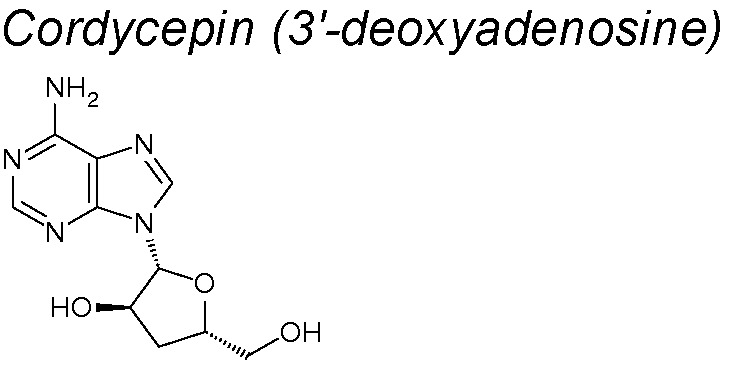	Upregulates E-cadherin and downregulates N-cadherin protein expression	*in vitro*	Su et al., 2017
7	Salvia miltiorrhiza	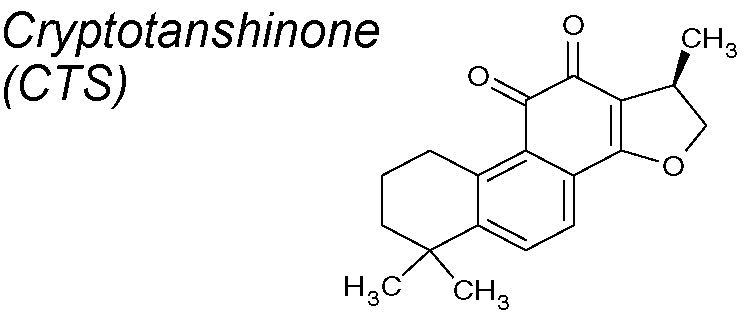	Inhibits TGF-β1/ Smad3/integrin β1 signaling pathway	*In vivo* and *in vitro*	Li et al., 2015; Zhu et al., 2016; Jin et al., 2013; Ma et al., 2012; Ma et al., 2014; Wang et al., 2017c
*7,13,15,26*	*Curcuma longa*	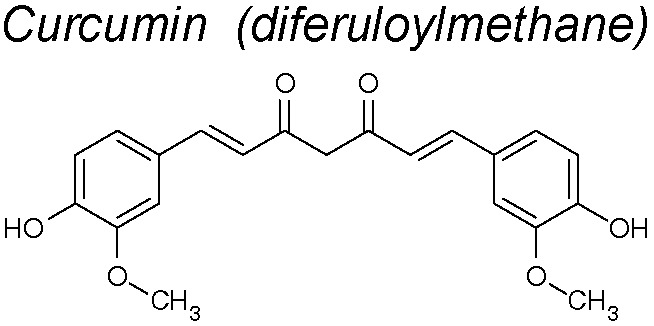	Blocks the PI3K/Akt/NF-κB signaling pathway	*In vitro*	Li et al., 2018b
			Suppresses the cav-1 phosphorylation stabilizating β-catenin	*In vivo and in vitro*	Sun et al., 2014
			Inhibits TGF-β/Smad signaling	*In vivo and in vitro*	Kong et al., 2015
*9,14,20,21*	*Jatropha isabelli* and *Jatropha gossypiifolia*	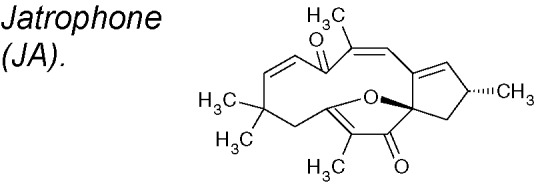	Inhibits Wnt/β-catenin signaling and reduces mRNA expression levels for SLUG, fibronectin and vimentin.	*In vitro*	Fatima et al., 2017
4,19,20,21	*Duchesnea indica* and* Duchesnea chrysantha*	*Duchesnea extracts.* Involve a wide range of chemical compounds.	Downregulates N-cadherin, fibronectin and vimentin and upregulates E-cadherin expression. Exerts antioxidant action.	*In vivo* and *in vitro*	Chen et al., 2017c; Kim et al., 2002b; Kim et al., 2007; Hu et al., 2009; Hu et al., 2011
*4,12,21*	Green tea leaves	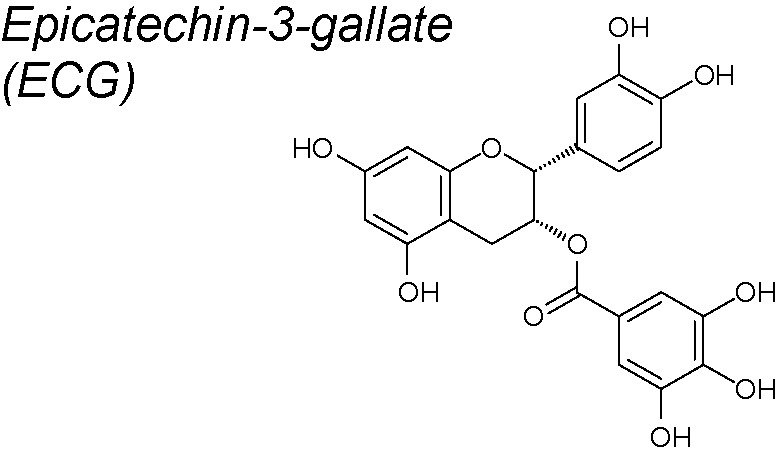	Downregulates fibronectin expression, inhibits p-FAK and upregulates E-cadherin expression	*In vivo* and *in vitro*	Huang et al., 2016
*3,12,23*	*Inula britannica*	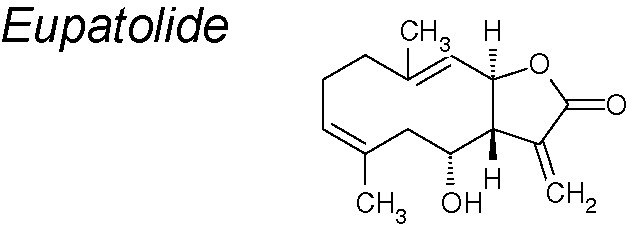	Suppresses TGF-β1-induced EMT via downregulation of Smad3 phosphorylation and decreasing the TGF-β type 1 receptor.	*In vitro*	Lee et al., 2010a; Kim et al., 2013; Wrighton et al., 2009; Boldbaatar et al., 2017
*3,4,9,19,20,21*	*Polygonum minus*	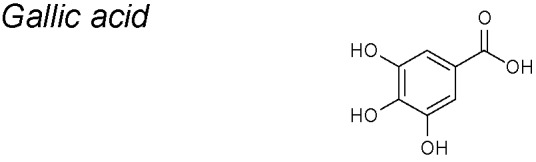	Downregulates collagen types I, III, fibronectin, CTGF, N-cadherin, vimentin, SNAI1, TWIST1 expression, and Smad3 phosphorylation	*In vitro* and *in vivo*	Kee et al., 2014; Ryu et al., 2016; Jin et al., 2017
*7*	*Garcinia hanburyi *Hook.f.	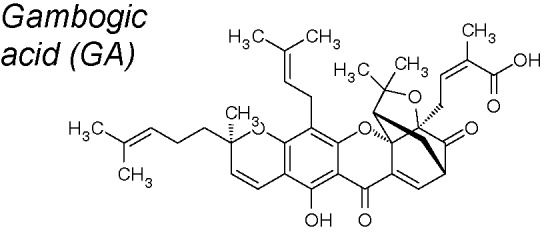	Suppresses TGF-β1/Smad3 pathway signaling and modulates VASH-2/VASH-1	*In vitro* and *in vivo*	Qu et al., 2016
*4,9,10,18,19,20*	*Azadirachta indica*	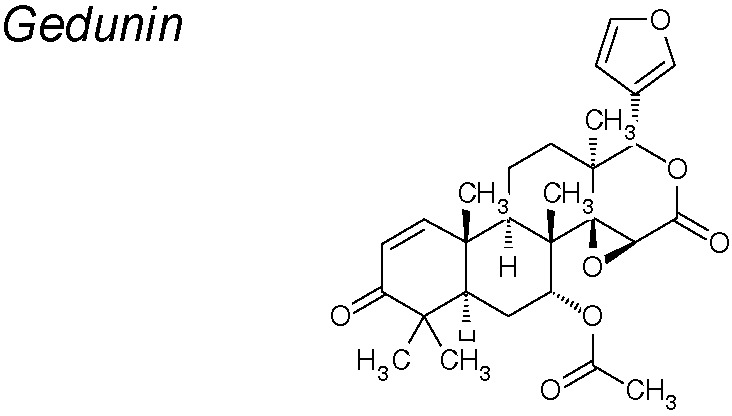	Decreases expression of N-Cadherin, Slug, Snail, Vimentin, Notch 1 and 2, and Zeb while increases expression of E-cadherin.	*In vivo* and *in vitro*.	Subramani et al., 2017
*7,4*	Soybeans	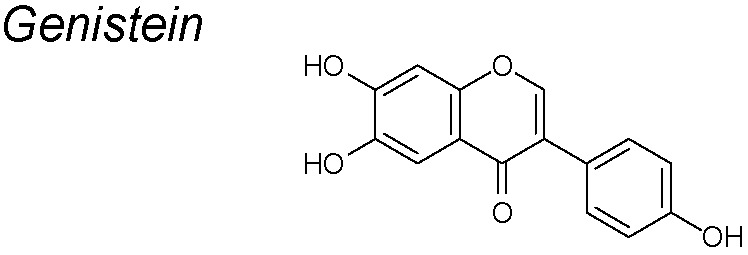	Downregulates TGF-β pathway signaling.	*In vitro*	Kim et al., 2015
			Blocks Smad4-dependent and independent pathways signaling through p38 MAPK	*In vitro*	Han et al., 2012
			Downregulates the nuclear factor of activated T cells 1 (NFAT1)	*In vitro*	Dai et al., 2015
*7*	*Gentianae*	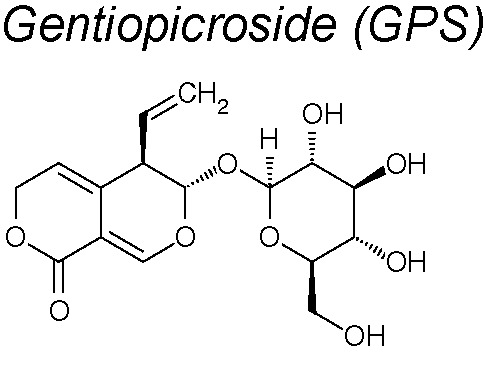	Downregulates the expression of TNF-alpha, IL1-b, TGF-β1 and CTGF	*In vivo* and *in vitro*	Chen et al., 2017b
*12,19,20,23*	*Dendrobium draconis*	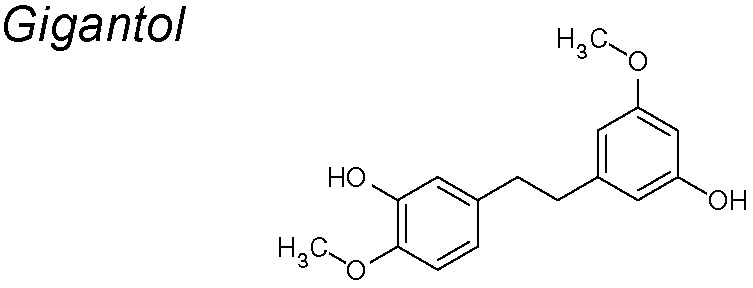	Downregulates N-cadherin, vimentin, and Slug, Inhibits AKT, ERK, and caveolin-1 (cav-1) signaling	*In vitro*	Unahabhokha et al., 2016
*14,15*	*Panax quinquefolius* and *notoginseng*	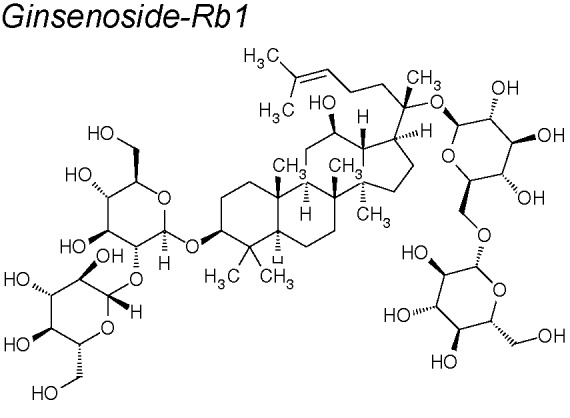	Inhibits Wnt/β-catenin signaling and EMT	*In vitro*	Deng et al., 2017
*4,5,9,19,24*	*Magnolia* spp. (officinalis, obovata, and grandiflora)	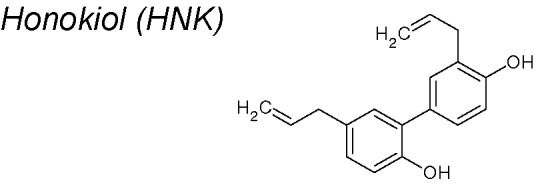	Downregulates Stat3 and Zeb1 expression. Upregulates E-cadherin	*In vitro* and *in vivo*	Avtanski et al., 2014
			Downstream pathways of c-FLIP are NF-κB signaling and N-cadherin/snail signaling		Lv et al., 2016
*7*	*Ligusticum wallichii* Franchat.	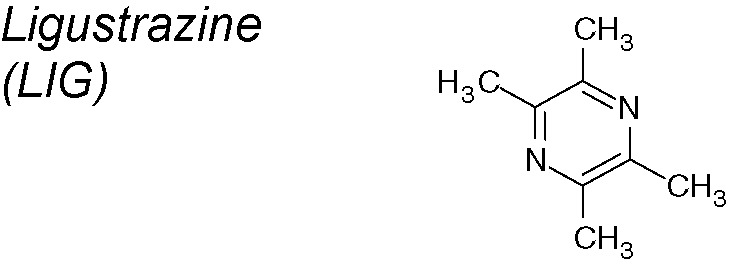	Downregulates the TGF-β1-induced loss of cytokeratin-18 expression.	*In vivo*	Yuan et al., 2012
*15,18*	Naturally found in several plant species including *Lonicera japonica* (Caprifoliaceae)	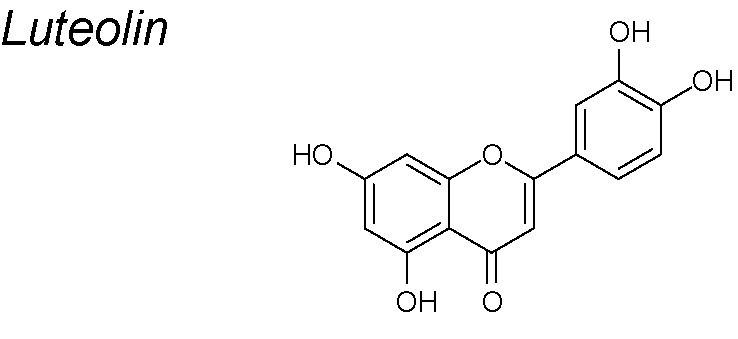	Suppresses Notch1 signaling	*In vitro*	Zang et al., 2017
			Downregulates β-catenin expression. Upregulates epithelial markers (E-cadherin and claudin) while downregulates mesenchymal markers (N-cadherin, vimentin, Snail and Slug).	*In vitro* and *in vivo*	Lin et al., 2017
*9,10,13,18,19,20,23,25*	*Azadirachta indica*	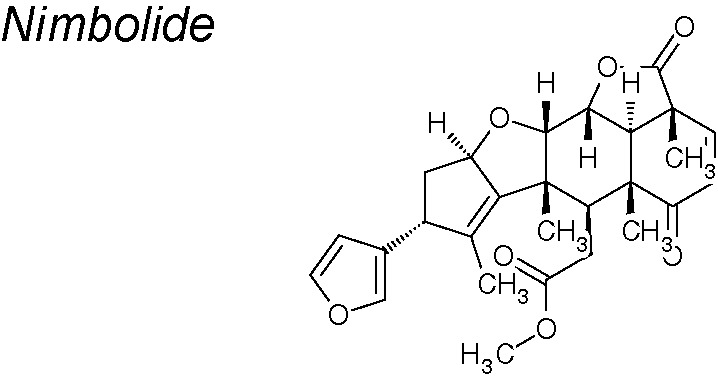	Reduces PI3K/AKT/mTOR and ERK signaling and decreases Notch-2, N-cadherin, vimentin and Snail, Slug and Zeb expression	*In vitro*	Bodduluru et al., 2014; Hao et al., 2014; Subramani et al., 2016
*4,8,9,10,19,20*	*Zanthoxylum nitidum*	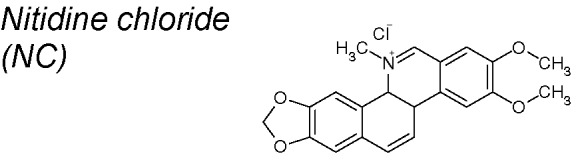	Inhibits cellular migration and invasion. Downregulates Snail, Slug and Zeb1, decreases N-cadherin and Vimentin and increases E-cadherin expression	*In vitro*	Sun et al., 2014; Sun et al., 2016
*4,7,10*	*Cnidium monnieri*	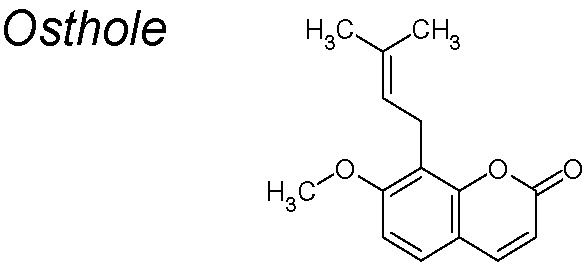	Inhibits the TGF-β/Akt/MAPK pathways signaling, reduces Snail-DNA-binding activity and induces E-cadherin expression	*In vivo* and *in vitro*.	Wen et al., 2015
*7*	*Sophora japonica*	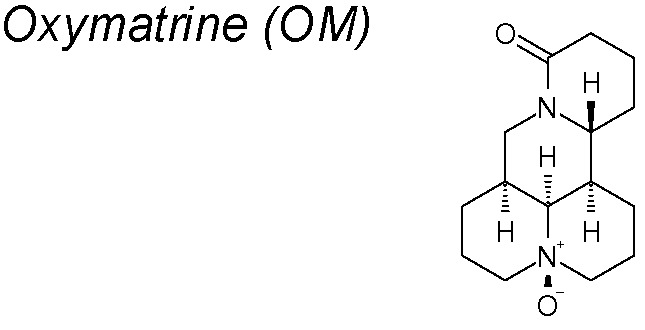	Blocks TGF-β1/Smad pathway signaling		Liu et al., 2012; Wu et al., 2008; Shi and Li, 2005; Chen et al., 2008; Shen et al., 2011; Fan et al., 2012; Liu et al., 2016
*3,7,9,10,16,19,20*	*Paeonia lactiflora* Pallas	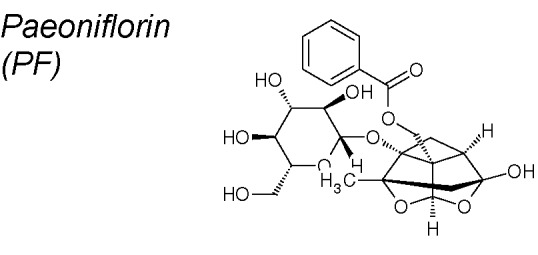	Downregulates TGF-β1 expression, maintains BMP-7 mRNA expression, and inhibits Smad2/3 activation	*In vivo*	Zeng et al., 2013
			Downregulates TGFβ, snail, N-cadherin, vimentin and MMP-2/-9 expressions	*In vivo* and *in vitro*	Wang et al., 2018
			Inhibits collagen-I synthesis and downregulates Snail and Slug expressions upregulating smad7	*In vivo*	Ji et al., 2016
*16,15,11,5,4*	*Paeonia suffruticosa* Andrews *(Cortex Moutan)*	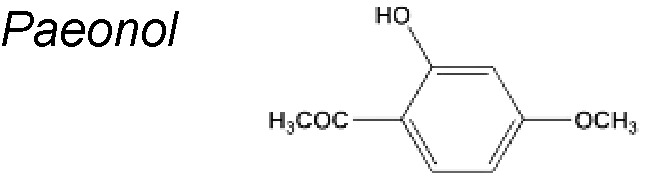	Decreased the expression levels of PCNA, β-catenin, p53, and COX-2. Upregulated E-cadherin and MMP-2/-9, also eliminates ROS	*In vivo* and *in vitro*	Lu et al., 2018; Zhang et al., 2015; Lin et al., 2014; Chou, 2003
*5*	*Tanacetum parthenium*	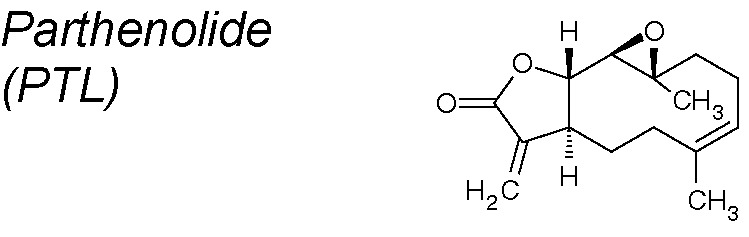	Blocks EMT via the NF-κB/Snail pathway	*In vitro* and *in vivo*	Hehner et al., 1999; Li et al., 2018a
*6*	*Ficus microcarpa*	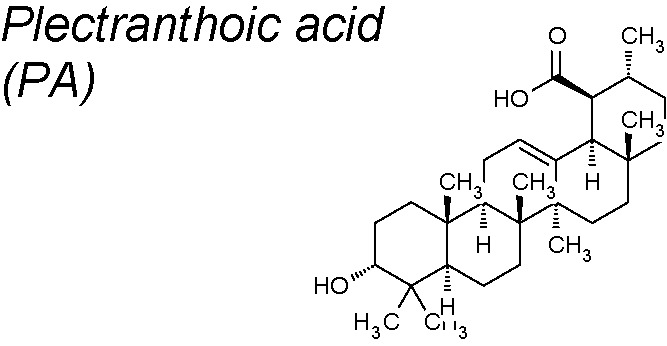	Inhibits NEDD9/Rac1 signaling	*In vitro*	Akhtar et al., 2018
*4,9,10,12,19,20*	*Dendrobium venustum*	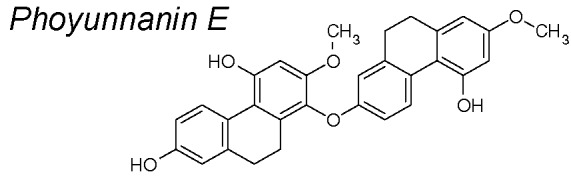	Suppresses FAK/AKT signals, decreases N-cadherin, vimentin, snail, and slug, and increases E-cadherin	*In vitro*	Petpiroon et al., 2017
*4,9,10*	*Piper longum*	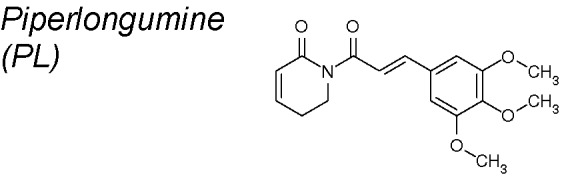	Downregulates the expression of Snail, Slug, β-catenin, zeb1, N-Cadherin, Claudin-1, and ZO-1	*In vivo* and *in vitro*	Liu et al., 2017
*1,9,10,11,15,20*	*Plumbaginaceae* plants	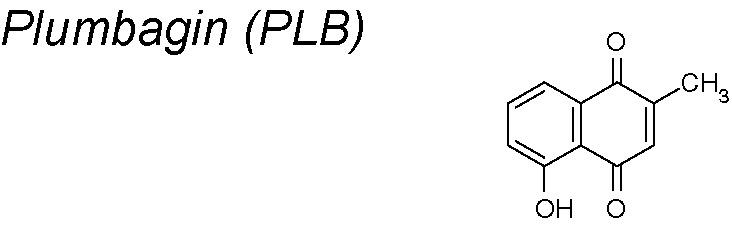	Inhibits Nrf2-mediated oxidative stress signaling pathway. Downregulates snail, slug, TCF-8/ZEB1, β-catenin, and vimentin and upregulates claudin-1 and ZO-1 expression.	*In vitro*	Pan et al., 2015
*17,24*	*Polyphylla rhizomes*	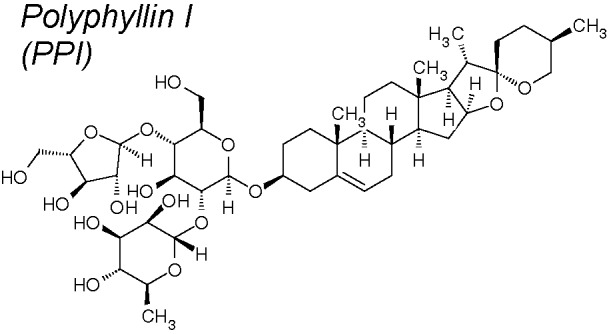	Blocks IL-6/STAT3 signaling pathway and stimulates epithelial marker expressions. Blocks EGF receptor tyrosine kinase inhibitors.	*In vitro*	Lou et al., 2017
*7,9,10,8,16*	Resveratrol (can be obtained from grapes, wine, mulberries and peanuts)	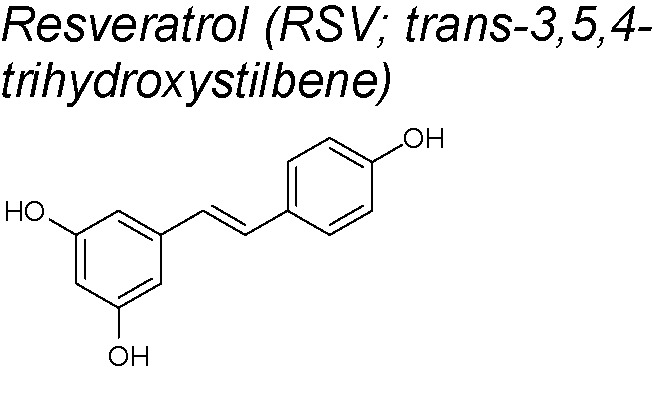	Suppresses TGF-β1-induced EMT, downregulates Snail and Slug expression, up-regulates E-cadherin and down-regulates fibronectin and vimentin	*In vitro*	Wang et al., 2013
			Inhibits the Hedgehog signaling pathway	*In vitro*	Bai et al., 2014b; Gao et al., 2015
			Upregulates SIRT1 and inhibits Smad4 and MMP7 expression	*In vivo* and *in vitro*	Xiao et al., 2016
			Suppresses MMP-2/-9 *via* MAPK and NFkb signals	*In vitro*	Liu et al., 2010a; Yang et al., 2009
			Represses EGF-induced ERK	*In vitro*	Vergara et al., 2011
			Downregulates Zeb-1, Slug and Snail.	*In vitro*	Shankar et al., 2011
11,22	*Salvia miltiorrhiza* Bunge	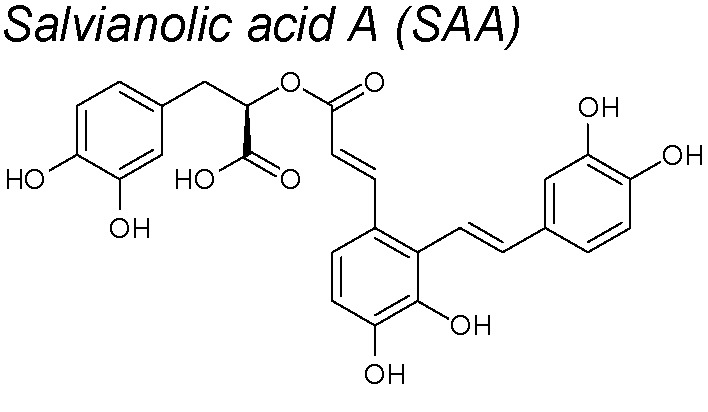	Downregulates α-SMA expression, suppresses oxidative stress. Inhibits the Nrf2/HO-1 pathway signaling	*In vivo* and *in vitro*	Chen et al., 2016a; Chen et al., 2017d
*8*	*Sedum sarmentosum* Bunge	*Sedum sarmentosum* Bunge (SSBE) extract	Downregulates the Hedgehog signaling activity.	*In vivo* and *in vitro*	Bai et al., 2014a; Bai et al., 2014b; Bai et al., 2017
*15*	Cruciferous plants “broccoli sprouts”	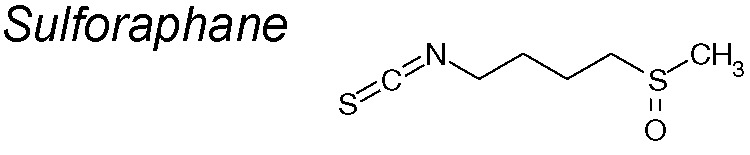	Blocks miR-616-5p/GSK3β/β-catenin pathway signaling	*In vivo* and *in vitro*	Wang et al., 2017a
*7,19,20,21*	Natural dietary polyphenolic compound	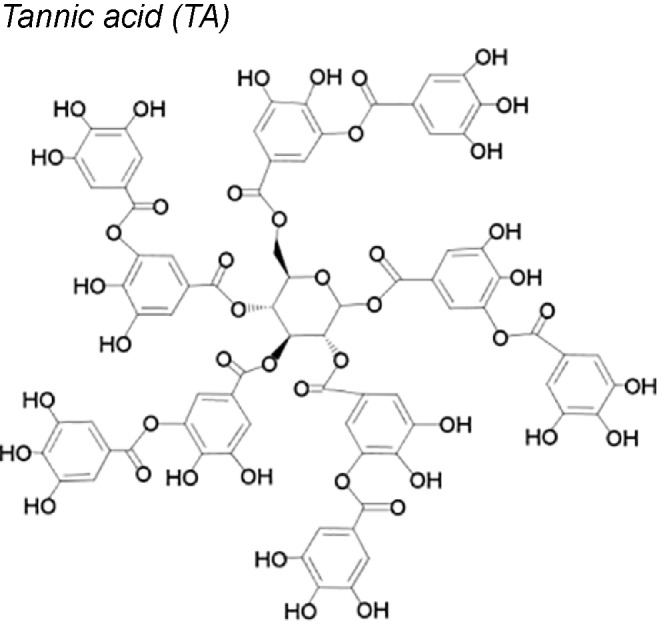	Reduces the TGF-β1-induced increase in TGF-β receptors expression. Decreases expression of N-cadherin, type-1-collagen, fibronectin, and vimentin.	*In vitro*	Pattarayan et al., 2018
*4,14,15*	*Withania somnifera*	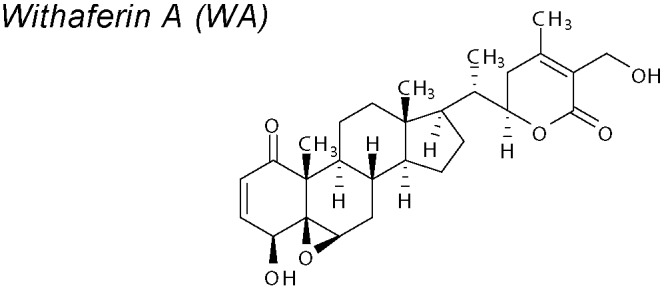	Witha-D partially inhibits EMT acting on Wnt/β-catenin signaling and recovering E-cadherin expression	*In vitro*	Chaurasiya et al., 2008; Sarkar et al., 2014
*4,16,20*	*Solanum nigrum* Linn.	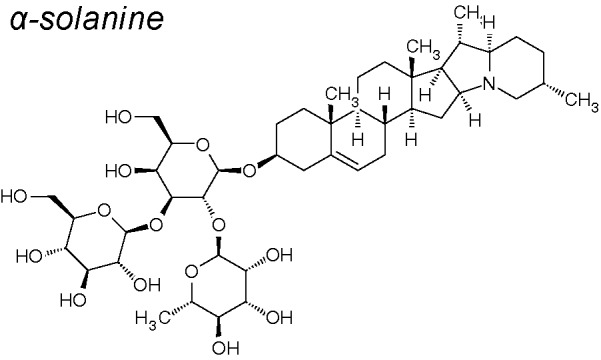	Reduces ERK and PI3K/Akt phosphorylation. Inhibits expression of MMP-2/-9, decreases vimentin, and increases E-cadherin.	*In vitro*	Shen et al., 2014

Chemical structures of the natural products included in this publication were obtained from scifinder and drawn with the program MarvinSketch.

***Arctigenin (ARC).*** It has been proposed as an anti-inflammatory and anti-cancer substance. In human lung cancer cells, ARC has been shown to inhibit TGF-β-induced phosphorylation, smad2/3 transcriptional activity, snail and N-cadherin expression, by contrast increasing the expression of E-cadherin in dose and time dependent manners. It blocks ERK-phosphorylation and β-catenin transcriptional activity. Through these mechanisms, ARC represses TGF-β-induced EMT (Xu et al., 2017).

***Baicalin and baicalein.*** These molecules significantly decreased the TGF-β1-mediated EMT, by reducing the Slug expression and NF-κB signaling pathway in mammary epithelial cells. Likewise, both molecules decremented growth and cell migration capacities of human breast cancer cells (Chung et al., 2015). In the same way, *baicalin* also inhibited SMADs 2 and 3 phosphorylation and suppressed migration and invasion in pancreatic cancer cells (Zheng et al., 2016).

***Berberine.*** It exhibits strong anti-cancer, anti-inflammatory, and anti-microbial effects (Tillhon et al., 2012). Kou et al. detected that berberine markedly upregulated E-cadherin and downregulated N-cadherin, fibronectin and vimentin expressions. Cadherin-bound β-catenin, which is required for cell adhesion, was also inhibited. The therapeutic espectrum of berberine also involved the downregulation of Snail, Slug and zinc finger E-box binding homeobox 1 (Zeb1) as well as the regulation of PI3K/Akt and retinoic acid receptor alpha and beta (RARα/RARβ) signaling, acting on the proliferation capacity of various cancer cells (Liu et al., 2015; Kou et al., 2016).

***Betanin.*** It presents powerful anti-oxidative and anti-inflammatory activities. Studies from Sutariya and Brijesh showed that betanin reduces streptozotocin (STZ) fibrosis induced in diabetic nephropathy model, by modulating EMT associated markers such as of TGF-β, type IV collagen, α-SMA and E-cadherin expression. Therefore, betanin can efficiently suppress renal fibrosis in diabetic nephropathy and may slow down the advancing to end-stage renal disease by regulating TGF-β pathway (Sutariya and Brijesh, 2017).

***Black rice anthocyanins (BRACs).*** These NPCs are extracted from the black rice, considered as a healthy food due to its effects on the liver and gastrointestinal tract (Kong et al., 2012). Anthocyanins happen to have potential beneficial effects such as antioxidant, anti-inflammatory, anti-cancerous and anti-metastatic effects (Hou, 2003; Sehitoglu et al., 2014). Zhou *et al*. observed that focal adhesion kinase (FAK) signaling pathway plays a function in the anti-metastatic properties of BRACs, decreasing the adhesion, migration and invasion of human HER-2-positive breast cancer cells *in vitro*. Likewise, these NPCs significantly modified the morphology of those cells from a mesenchymal to an epithelial phenotype. BRACs elevated the expression of E-cadherin and reduced the expression of fibronectin and vimentin (Zhou et al., 2017).

***Brusatol (BR).*** This NPC strongly inhibits pancreatic tumor action *in vitro* (Zhao et al., 2011). Research sugests that BR could sensitize the current first-line chemotherapeutic agents to pancreatic cancer *via* inhibition of the EMT process. It has been proven that BR increased E-cadherin while decreasing vimentin protein expressions, and also reducing Twist mRNA expression (Lu et al., 2017).

**Carnosol (CAR)** is a naturally occurring phenolic diterpene located in several Mediterranean herbs and is a main component of rosemary (*Rosmarinus officinalis *L). It has been reported that the CAR anti-proliferative actions is preferentially directed towards cancer cells, as reported in both animal and *in vitro* models. Furthermore, CAR presented a favourable therapeutic option in glioblastoma cells (Johanson, 2011; Vergara et al., 2014; Giacomelli et al., 2016). CAR could interfere with the diverse processes implicated in cancer resistance and aggressiveness, such as cancer stem cells (CSC) formation, proliferation and self-renewal. Fascinatingly, also diminished the influence of the cancer microenvironment by reducing the cytokine- induced EMT that underlies the possession of the mesenchymal phenotype. Likewise, CAR had the ability to reactivate the p53 functionality promoting CSC proliferation control and decreasing EMT was highlighted for the first time (Giacomelli et al., 2017).

It also possesses anti-cancer effects on several tumor types. It has shown to promote apoptotic cell death through p53 functional reactivation and to control the TNF-α/TGF-β-induced EMT, counteracting the effects of the cytokine on EMT master regulator genes (Slug, Snail, Twist and ZEB1). It has also been demonstrated that CAR is able to modulate the activation of miR-200c, a key player in the EMT process. Finally, CAR increase the temozolomide anti-proliferative effects *in vitro* (Giacomelli et al., 2017).

***Cairicoside E (CE).*** It has been published that this compound down-regulates the Aquaporin-5 (AQP5) expression and suppressed the EMT process in colon-rectal cancer cells. Research suggested that TGF-β1 increased the expression of AQP5 and activated the EMT byincreasing the expressions of p-Smad2/3, while silence of AQP5 with CE blocked the levels of p-Smad2/3 (Chen et al., 2017a).

***Celastrol.*** It is a pharmacologically active element that demonstrates significant therapeutic actions in chronic inflammatory, autoimmune, cancer, and neurodegenerative disorders (Allison et al., 2001; Dai et al., 2010; Ge et al., 2010; Venkatesha et al., 2011; Wong et al., 2012). Recently, Divya et al. suggested that celastrol decreased the N‐cadherin, snail, slug, vimentin and β‐catenin expression in a Bleomycin‐induced lung fibrosis rat model. They, likewise, feature this anti-EMT effect to the inhibition of heat shock protein 90 inhibition (Divya et al., 2018). Other research established that celastrol suppresses inflammatory reactions, as well as regulates oncogenic proteins including β-catenin. Celastrol also decreases pro-inflammatory cytokines (TNF-α, IL-1β and IL-6) serum concentrations, down-regulates cyclooxygenase 2 (COX-2), inducible nitric oxide synthase (iNOS), N-cadherin, Vimentin and Snail expressions, inactivates NF-κB and upregulates E-cadherin (Lin et al., 2015).

***Celastrus orbiculatus Thunb extract (COE).*** The extracts from the stems of this plant constitute 11 compounds (Li et al., 2012a). The ethyl acetate of COE constrains the proliferation, EMT (targeting VEGF, activating MAPK and inhibiting Akt signaling pathways), invasion and metastatic faculties of tumor cells (Qian et al., 2012; Zhang et al., 2012a). Moreover, COE is used in the antiinflammatory and analgesic handling of various diseases. In human gastric cancer AGS cells, it has been observed that Cofilin 1, Ncadherin, vimentin, MMP2 and MMP9 proteins expressions were significantly reduced by COE, whereas the Ecadherin expression was increased (Wang et al., 2017b).

***Cinnamomum cassia extracts (CCE).*** Fifteen compounds were isolated from the bark extract of *C. cassia* growing in China (Zhao et al., 2013). These extracts were shown to diminish the TGF-β1-induced motility and invasive capacities of A549 and H1299 cells by inhibiting MMP-2 and urokinase-type plasminogen activator. Moreover, they impaired cell adhesion associated with collagen production. CCE also down-regulated vimentin and fibronectin and upregulated E-cadherin expressions (Lin et al., 2017).

***Codonolactone (CLT).*** It inhibited the expression of acquired EMT’s mesenchymal markers such as N-cadherin and vimentin in a dose-dependent manner in *in vivo* and *in vitro* experiments in breast cancer. Likewise, it blocked the expression of transcription factors Snail, Slug, Twist-1 (TGF-β signaling) and the Runx2 phosphorylation (Fu et al., 2016).

***Cordycepin.*** Its properties have been evaluated on a human oral squamous cell carcinoma xenograft model, through its administration in a regular, low-dose upregulated E-cadherin and downregulated N-cadherin protein expressions, inhibiting EMT (Su et al., 2017).

***Cryptotanshinone (CTS).*** It exhibits multiple pharmacological benefits, involving anti-cancer (Li et al., 2015; Zhu et al., 2016), anti-oxidative stress (Jin et al., 2013), and anti-cardiac fibrosis properties *via* downregulation of COX-2, NADPH oxidase 2 and 4 and MMP-2 (Ma et al., 2012; Ma et al., 2014). The antifibrotic mechanism proposed for CTS is the inhibition of Smad2 phosphorylation. Although it did not inhibit Smads 3 and 4 or mitogen-activated protein kinase (MAPK) signals, the ECM accumulation was importantly reduced in a renal fibrosis model (Wang et al., 2017c).

***Curcumin.*** This NPC inhibit EMT in CoCl_2_-treated hepatocytes. This action might be due to its capacity to diminish TGF-β-R1 expression in these cells. This effect suggests a disruption on the downstream signal transduction transmitted by SMADs pathway (Kong et al., 2015). Moreover, it was found that SMADs2 and 3 phosphorylation was inhibited by curcumin, pointing its powerful action blocking upstream the EMT pathway signaling. Another mechanism by which curcumin inhibits these pathways is by suppressing the cav-1 phosphorylation, stabilizating β-catenin (Sun et al., 2014). Recently, it has been published that curcumin inhibits the superoxide dismutase-induced invasion and migration of pancreatic cancer cells by inhibiting the PI3K/Akt/NF-κB signaling pathway (Li et al., 2018b). Curcumin was not related to toxicity including high dose administration, in human clinical trials (Gupta et al., 2013; Hewlings and Kalman, 2017).

***Jatrophone (JA)*** **.** This diterpene shows a broad assortment of biological actions, counting antitumoral, cytotoxic, anti-inflammatory, anti-malarial and fungicidal properties (Devappa et al., 2010). It has been demostrated that JA reduces mRNA expression of Slug, fibronectin and vimentin, but not ZEB1, and also exhibits an anti-proliferative and anti-migratory effect acting on Wnt/β-catenin signaling in triple-negative breast cancer (Fatima et al., 2017).

***Duchesnea extracts.*** *Duchesnea chrysantha* and *Duchesnea indica* belong to the *Rosaceae* family, and their extracts show a diversity of biological properties, involving anti-biotic, anti-oxidative, anti-inflammatory and some cytotoxic features (Kim et al., 2002b; Kim et al., 2007). These extracts involve a range of chemical compounds such as triterpenes, triterpene glycodides, flavonoid glycodides and sterols (Lee et al., 1994; Qiao et al., 2009). An *in vivo* research showed that tumor growth was importantly diminished in BALB/c nude mouse xenograft model orally treated with *Duchesnea indica *extracts (DIE) (Chen et al., 2017c). In the same study, DIE also inhibited highly metastatic cells by reducing the secretions of MMP‐2 and urokinase‐type plasminogen activator (uPA) (Chen et al., 2017c). It was also able to decrease the cell adhesion capacity, down-regulate the N-cadherin, fibronectin, and vimentin and increase the E-cadherin expression (Kim et al., 2002b; Kim et al., 2007; Chen et al., 2017c). Another protective mechanism related with DIE is its antioxidant action which can also modulate the EMT (Hu et al., 2009; Hu et al., 2011).

***Epicatechin-3-gallate (ECG)*** **.** It elicits several anti-oxidant and anti-inflammatory activities and is one of the four types of catechins mainly detected in green tea, together with epicatechin, epigallocatechin and epigallocatechin-3-gallate (EGCG) (Chowdhury et al., 2016). In human lung cancer cells, ECG also reverts the TGF-β1-induced EMT by upregulating epithelial markers (E-cadherin) and downregulating mesenchymal markers (fibronectin). Moreover, it also phosphorylates FAK. Based on these facts, it has been recommended that ECG may be administered as an effective agent against TGF-β1-induced EMT (Huang et al., 2016).

***Eupatolide*** **.** It shows anti-inflammatory, anti-proliferative and anti-migratory effects (Lee et al., 2010a; Kim et al., 2013). It has also been suggested that eupatolide might be employed as an inhibitor of the TGF-β1 signaling pathway to suppress EMT (Wrighton et al., 2009). Moreover, eupatolide suppress TGF-β1-induced EMT *via* downregulation of Smad3 phosphorylation and decreasing the TGF-β type 1 receptor (Boldbaatar et al., 2017).

***Gallic acid.*** *In vivo* experiments with this NPC diminished vascular calcification, cardiac hypertrophy, cardiac fibrosis and hypertension. Gallic acid also inhibited pathological changes in the lungs, such as pulmonary fibrosis (Kee et al., 2014; Ryu et al., 2016). Moreover, it reduced the expression of fibrosis-related genes, including collagen types I and III, fibronectin, connective tissue growth factor (CTGF), and Smad3. In a mouse model, Garlic acid blocked the of EMT-related genes expression, such as N-cadherin, vimentin, Snail, and TWIST1 (Jin et al., 2017).

***Gambogic acid (GA).*** It has been proved *in vitro* that this compound reverses TGF-β1-mediated EMT and endothelial–mesenchymal transition (EndoMT) in human lung fibroblasts (HLF-1). It also prevents pulmonary fibrosis *in vivo* and attenuates the EMT by modulating the TGFβ1/Smad3 pathway (Qu et al., 2016).

***Gedunin.*** It has been shown to have potential anti-cancer activity (Kamath et al., 2009; Patwardhan et al., 2013; Hao et al., 2014). Recent research suggests that gedunin inhibits EMT by reducing the expression of the mesenchymal markers N-Cadherin, Slug, Snail, Vimentin, Notch 1 and 2, and Zeb whereas increasing the E-cadherin expression (Subramani et al., 2017).

***Genistein (GEN)*** **.** Soybeans and most soy products contain large amounts of isoflavones called soy phytoestrogens, and one of the most concentrated is the GEN (Lee et al., 2012). GEN is a phytoestrogen known for its chemopreventive effects in several types of cancers (Kim et al., 2015). It suppresses the EMT response induced by 17β-estradiol and two estrogens-like compounds, bisphenol-A and nonylphenol. Thus, it reduces the protein expressions of vimentin, cathepsin D, and MMP-2, increases E-cadherin expression and downregulates TGF-β. (Kim et al., 2015). In ovarian cancer derived cells, GEN inhibits the NF-κB and *Akt* signaling pathways, playing important roles in keeping the homeostatic balance between cell survival and apoptosis. It has been considered as a potencial antiangiogenic, antioxidant and anticancer agent (Han et al., 2012; Dai et al., 2015).

***Gentiopicroside (GPS).*** It has been proved that in bronchoalveolar cells isolated from fluids of lungs pulmonary fibrosis in a mouse model, GPS decreased the levels of proinflammatory cytokines, including TNF-α and IL-1β, and downregulated TGF-β1 and CTGF expression. *In vitro*, GPS inhibited the EMT of A549 cells stimulated by TGF-β1 to induce transdifferentiation at a dose-dependent manner (Chen et al., 2017b).

***Gigantol.*** It has been described to have anti-proliferative, anti-apoptosis and anti-metastatic properties (Charoenrungruang et al., 2014; Klongkumnuankarn et al., 2015). Recent publications suggest that gigantol considerably reduces lung cancer cells’ viability in a detached condition. It also shrinkages EMT biomarkers including N-cadherin, vimentin and Slug, leading to a meaningful suppression of AKT, ERK, and cav-1 survival pathways (Unahabhokha et al., 2016).

***Ginsenoside*** **.** It has been registered in pharmacopeias for thousands of years due to its abundant content of saponins. One of the most extensively known saponins in the rhizome of ginseng is ginsenoside-Rb1 (Jia and Zhao 2009). *In vivo* studies mention that Rb1 showed cardioprotective, hepatoprotective and anti-inflammatory effects (Wang et al., 2008; Li et al., 2012b; Cheng et al., 2013; Hou et al., 2014). Likewise, it inhibits cell proliferation, angiogenesis and apoptosis stimulation (Zheng et al., 2013; Lee et al., 2016). A recent publication shows that ginsenoside-Rb1, especially its metabolite compound K, particularly sensitize cancer stem/tumor-initiating cells from ovarian cancer to chemotherapy through the inhibition of Wnt/β-catenin signaling and EMT (Deng et al., 2017).

***Honokiol (HNK)*** **.** It has been associated with anti-tumor and more recently anti-EMT effects (Fujita et al., 1973; Lee et al., 2005; Ahn et al., 2006; Sheu et al., 2008; Arora et al., 2011; Arora et al., 2012; Nagalingam et al., 2012). For instance, in breast cancer cells, Avtanski et al. demonstrated that HNK inhibited signal transducer and activator of transcription 3 (Stat3) phosphorylation and transactivation activity and Zeb1 expression, which plays a main role in EMT initiation. More than that, HNK induces an increase in E‐cadherin (Avtanski et al., 2014). Additionally, it has been published that HNK inhibits EMT motility and migration by targeting cellular FLICE (FADD-like, IL-1β-converting enzyme)-inhibitory protein (c-FLIP), considered a master anti-apoptotic regulator in non-small-cell lung cancer (Lv et al., 2016).

**Isoviolanthin** extracted from the leaves of *Dendrobium officinale* inhibits transforming growth factor (TGF)-β1-induced EMT in hepatocellular carcinoma (HCC) cells, it is the most significant constituents responsible for the antimetastasis activity of *Dendrobium officinale*. Recent publications report that isoviolanthin targets the TGF-β/Smad and PI3K/Akt/mTOR pathways to repress TGF-β1-induced EMT phenotypes in HepG2 and Bel-7402 HCC cells. Furthermore, these results confirm that isoviolanthin could be a favorable natural compound with low toxicity for the treatment of metastatic HCC by affecting TGF-β1-induced EMT (Xing et al., 2018).

***Ligustrazine (LIG).*** In a model of renal tubulointerstitial fibrosis, LIG showed pleyotropic effects acting at different levels of EMT induction. LIG ​​decreased the mRNA expression of TGF-β1, CTGF, monocyte chemoattractant protein-1 (MCP-1) and osteopontin, and, subsequently cytokeratin-18 expression decreased. Mainly, this molecule increased the expression of the natural inhibitors of TGF-β, hepatocyte growth factor (HGF) and bone morphogenetic protein (BMP)-7 (Yuan et al., 2012 **)**.

***Luteolin*** **.** Many biological properties of luteolin, such as anti-inflammation, anti-allergy, antioxidant, anticancer and anti-microbial effects have been described (Chung et al., 2001; Chen et al., 2007; Lin et al., 2008). In breast cancer (*in vivo* and *in vitro*), epithelial markers such as E-cadherin and claudin were upregulated in response to luteolin while mesenchymal markers N-cadherin, vimentin, Snail and Slug were downregulated at dose-dependent manner. Researchers found that these positive effects of luteolin were extinguished by overexpression of β-catenin, indicating that downregulation of β-catenin expression may mediate the inhibitory effects of luteolin on EMT (Lin et al., 2017). Same results were found by Zang et al., who described that other pathways such as Notch1 were also blocked by Luteolin (Zang et al., 2017).

***Nimbolide*** **.** Recent studies indicate that treatment with this agent reduces the expression of Notch-2, N-cadherin, vimentin and transcription factors (Snail, Slug and Zeb) in pancreatic cancer cell lines. Moreover, nimbolide treatment likewise increased the expression of E-cadherin. Additionally, the generation of ROS mediated by nimbolide reduces cell proliferation (via reduction of PI3K/AKT/mTOR and ERK signaling) and metastasis (via reduction of EMT, invasion, migration and colony forming abilities) through mitochondrial-mediated apoptotis but not through autophagy (Bodduluru et al., 2014; Hao et al., 2014; Subramani et al., 2016).

***Nitidine chloride (NC)*** **.** It has been shown to exert antimalarial (Bouquet et al., 2012), anti-inflammatory (Wang et al., 2012), anti-angiogenic (Chen et al., 2012), and anticancer effects (Fang et al., 2013). Likewise, NC inhibited the cellular migration and invasion through suppression of FAK-associated pathway in breast cancer metastasis (Sun et al., 2014). It has also been recently proposed that inactivation of Hedgehog signaling pathway by NC led to significantly decreased Smo and Gli expressions, targeting breast cancer metastasis. Thus, NC could be suitable for the prevention and treatment of breast cancer through dual-blocking EMT (Sun et al., 2016).

***Osthole*** **.** It inhibits growth and metastasis in many types of cancer (Kao et al., 2012; Zhang et al., 2012b; Ding et al., 2014). It has also been proposed that osthole mediated the EMT by downregulating Snail and cell-invasive capability, suppressing the TGF-β/Akt/MAPK pathway (Wen et al., 2015).

***Oxymatrine (OM).*** Many studies have proved that OM shows an anti-fibrotic effect on liver, pulmonary, myocardial and skin scar tissue fibrosis through inhibition of the TGF-β1/Smad signaling cascade (Shi and Li, 2005; Chen et al., 2008; Wu et al., 2008; Shen et al., 2011; Fan et al., 2012; Liu et al., 2012). Thus, Liu *et al*. demonstrated that OM inhibits the high glucose-induced renal tubular EMT, decreasing the degradation of SnoN mediated by a E3 ubiquitin ligase (Arkadia), and that promotes EMT amplifying TGF-β signalling through Smad7 degradation (Liu et al., 2016).

***Paeoniflorin (PF)*** **.** Pharmacological reports have shown that it prevents pulmonary EMT inhibiting collagen type-I synthesis, downregulating Snail and Slug and up-regulatining Smad7. These properties provide PF a protective action against cellular transdifferentiation *i*n a lung bleomycin-induced fibrosis model in mice (Ji et al., 2016). It has also been demonstrated that PF down-regulates TGF-β1, maintains BMP-7 expression and inhibits Smad2/3 in a renal fibrosis model (Zeng et al., 2013). Likewise, PF blocks EMT in gliblastoma cells, and reduces TGF-β, Snail, N-cadherin, Vimentin and MMP2/9 expression at doses depended manner (Wang et al., 2018).

***Paeonol.*** It is an aspirin analogue extracted from numerous medicinal herbs including *Moutan Cortex, Cynanchi paniculati Radix et rhizome*, and *Paeoniae Radix rubra*. Paeonol was discovered to present comprehensive pharmacological activities, such as antioxidant, anti-inflammatory, anti-aging, and anti-cancer activities (Chou, 2003; Zhang et al., 2015). Another author reported that paeonol influenced antioxidative stress activity in endothelial cells by controlling the expressions of Sirt1. It has been described, too, that paeonol ameliorated colitis related colorectal cancer by suppressing cytokine-induced EMT and NF-jB activation (Lin et al., 2014). Likewise, suggested that paeonol inactivated ERK and TGF-beta1/Smad pathway leading to regulation of relevant EMT markers. These results suggest that paeonol might be developed as a potential agent used for oxidative stress injury and EMT in premalignant lesion (Yang et al., 2018).

***Parthenolide (PTL).*** It has been conventionally used for the treatment of headaches and arthritis. Recent analyses suggest that PTL is a valuable antitumor and anti-inflammatory NPC, and it was evaluated in clinical studies for leukemia and neurological tumors (Ghantous et al., 2013). These effects of PTL in tumors and inflammatory diseases primarily happen *via* the inhibition of NF-κB signaling pathways (Hehner et al., 1999). Current studies have established that PTL inhibit pulmonary fibrosis increasing E-Cadherin and decreasing vimentin NF-κB and Snail expression in TGF-β1-treated primary lung epithelial cells (Li et al., 2018a).

***Plectranthoic acid (PA)*** **.** It induces cell cycle arrest and apoptosis in prostate cancer cells (Akhtar et al., 2016). Recent research demonstrates that PA-exposed cells exhert considerably reduced cell migration capacity and a reversal of TGF-β induced EMT, representing the potential effectiveness of PA against prostate cancer, throughout regulation of Rac1 signaling (Akhtar et al., 2018).

***Phoyunnanin-E*** **.** Recent publications suggest that Phoyunnanin E decreased the E-cadherin to N-cadherin switch and reduced upregulation of mesenchymal markers such as vimentin and snail, as well as slug expression. Phoyunnanin-E has also been shown to inhibit migration and growth and promote EMT suppression, reduce migratory-associated integrins αv and β3, and suppress FAK/AKT cascade, which subsequently suppressed downstream migratory proteins in lung cancer cells (Petpiroon et al., 2017).

***Piperlongumine (PL)*** **.** It has been identified as a powerful cytotoxic element highly selective to cancer cells (Raj et al., 2011; Bezerra et al., 2013; Liu et al., 2014; Zheng et al., 2016; Zhou et al., 2016). PL has also been demonstrated to accurately suppress bladder cancer development both *in vitro* and *in vivo*, *via* inhibition of EMT. Thereby, the expression of EMT-associated factors such as Slug, β-catenin, zeb1, N-Cadherin, Claudin-1, and zonula occludens-1 (ZO-1) were importantly decreased (Liu et al., 2017).

***Plumbagin (PLB)*** **.** It presents anti-inflammatory, anti-atherosclerotic, anti-bacterial, anti-fungal, and anti-cancer properties shown both *in vitro* and *in vivo* (Padhye et al., 2012). The anti-EMT effect of the PLB can be vinculated by its ability to adjust epithelial adherent junctions in human tongue squamous carcinoma cells. PLB also boosted the expression of E-cadherin and decreased of N-cadherin in these cells. Moreover, it reduced the expression of Snail, Slug, TCF-8/zeb1, β-catenin, and vimentin, whereas increased the expression of claudin-1 and ZO-1. Notably, PLB inhibited the translocation of nuclear factor erythroid 2-related factor (Nrf2) from cytosol to nucleus, causing an inhibition in the expression of downstream targets (Pan et al., 2015).

***Polyphyllin (PP) I*** **.** It has been broadly investigated for its anti-inflammatory and anti-cancer activities. PPI exhibited inhibitory effect on various cancer types, involving hepatocarcinoma (Ong et al., 2008), non-small cell lung cancer (Kong et al., 2010), osteosarcoma (Chang et al., 2015), chronic myeloid leukemia (Wu et al., 2014), ovarian cancer (Gu et al., 2016) and glioma cells (Yu et al., 2014). Recent investigation described that PPI was capable to reverse EMT in osteosarcoma cells (Chang et al., 2015). Likewise, ZH-2, a compound derived from PP VII, exherts anti-chemoresistance properties through inhibiting EMT (He et al., 2016). In anacquired-erlotinib-resistant cell line, PPI inhibited IL-6/STAT3 signaling pathways and stimulates epithelial marker expression, reversing EMT. Significantly, PPI exhibited an inhibitory effect on epidermal growth factor (EGF) receptor tyrosine kinase inhibitors, which has a mutagenic and pro-EMT action in non-small cell lung cancer (Lou et al., 2017).

***Resveratrol (RSV)*** **.** RSV has been published to have many pharmacological activities, such as protection against coronary heart disease, anti-inflammatory properties, chemo-prevention of cancer, anti-oxidative and antiasthmatic effects (Frémont, 2000; Wallerath et al., 2002; Aggarwal et al., 2004; Bisht et al., 2010). It can be obtained from grapes, wine, mulberries and peanuts (Shakibaei et al., 2009). It also reduced renal injury and renal fibrosis by suppressing the inflammatory activity and by inhibiting lipid peroxidation (Chander and Chopra, 2005; De Jesus et al., 2007). The inhibitory effect of RSV on EMT has been demostrated in prostate (Li et al., 2014), ovarian (Baribeau et al., 2014),**breast (Tsai et al., 2013) and pancreatic cancer (Li et al., 2013). Recent papers show that RSV inhibits EMT in renal tubular cells by antagonizing the hedgehog signaling pathway (Bai et al., 2014b). Likewise, Gao *et al.* suggested that RSV prevents from cancer cell invasion and metastasis *in vitro* by inhibiting the hedgehog pathway and EMT (Gao et al., 2015). In this context, RSV downregulates the EMT-inducting transcription factor (including Zeb-1, Slug and Snail) to reduce migration and invasion in pancreatic cancer cells (Shankar et al., 2011). EGF is a well-known EMT-inducer in human breast cancer cells (Ackland et al., 2003; Vergara et al., 2011). RSV blocks EGF-induced EMT by repressing EGF-induced ERK (Vergara et al., 2011). Furthermore, it is known that renal injury has a close relationship with the development of renal fibrosis and, during this process, tubular epithelial cells in the kidney undergo EMT *via* upregulating β-catenin/lymphoid enhancer-binding factor 1 (LEF1) signaling and MMP-7 (Liu, 2004; Shibata et al., 2009). A current study using RSV showed that this product attenuated renal injury and fibrosis through inhibition of EMT. Authors suggested that this inhibition was due to the fact that RSV up-regulated sirtuin 1 (SIRT1), which deacetylated Smad4 and inhibited the expression of MMP-7 (Xiao et al., 2016). Other findings also demonstrate that RSV modulates EMT by suppressing MMP-2 and MMP-9 *via* MAPK and NF-κb signals in lung cancer invasion and metastatic cells (Yang et al., 2009; Liu et al., 2010a). Moreover, RSV has been recently shown to limit EMT by controlling gene expression at post-transcriptional level (it favors the epithelial-type alternative splicing of pre-mRNAs that encode crucial factors in adhesion and migration, and enhances the expression of some RNA-Binding Proteins) (Moshiri et al., 2017). It also inhibits TGF-β1-induced EMT and suppresses lung cancer invasion and metastasis (Wang et al., 2013)

***Salvianolic acid A (SAA)*** **.** It exerts many pharmacological actions, such as myocardial protection, anti-thrombosis, anti-fibrosis, and the prevention of diabetes complications (Ho and Hong, 2011; Xu et al., 2014). Investigations have revealed that SAA treatment effectively decreased lung parenchymal injury and collagen deposition and diminished the apoptosis and lung fibrosis on a pulmonary arterial hypertension rat model. Furthermore, in pulmonary tissue, SAA treatment upregulated BMP type II receptor (BMPRII) expression and augmented the Smad1/5 phosphorylation. Both molecules showed an anti-EMT effect (Chen et al., 2016a). An anti-EndoMT capacity was also discovered in bleomycin-induced pulmonary fibrosis in mice, acting on Nrf2/HO-1 signaling pathway (Chen et al., 2017d).

***Sedum sarmentosum Bunge (SSBE)*** **.** Pharmacological reports have shown that SSBE has significant antiinflammatory, anti-tumor and anti-angiogenic effects (Oh et al., 2004; Morikawa et al., 2007; Ninomiya et al., 2007; Jung et al., 2008). Other authors demonstrated that SSBE has marked effects against renal fibrosis (Bai et al., 2014a; Bai et al., 2014b), down-regulating hedgehog signaling pathway (which promotes renal fibrogenesis fostering the formation of myofibroblasts from different cell types through an EMT process). SSBE also reduced the ECM accumulation and angiogenesis (Bai et al., 2017).

***Sulforaphane.*** Numerous studies have observed the effects of *sulforaphane* in control of tumor generation or cancer progression, such as in lung, breast and prostate (Amjad et al., 2015; Atwell et al., 2015; Jiang et al., 2016), and also digestive system neoplasms (Jeon et al., 2011; Kim et al., 2015). Other authors demonstrated that reduced expression of the micro RNA miR-616-5p, transcriptionally induced by sulforaphane management, contributes to the suppression of EMT in non-small cell lung cancer and in lung cancer metastasis through the miR-616-5p/GSK3β/β-catenin signaling pathway (Wang et al., 2017a).

***Tannic acid (TA).*** This molecule acts upstairs in the EMT induction process, in lung epithelial cells. It reduces the expression of TGF-β and N-cadherin and decreases the SMADs 2 and 3 phosphorylation and the production of ECM (fibronectin and vimentin). Moreover, cell proliferation in G0/G1 phase and the mitogenic activity of protein kinase (ERK1/2, JNK1/2, and p38) also decrease (Pattarayan et al., 2018).

***Withaferin-A (WA)*** **.** Pharmacological reports have shown anti-cancer effects in rodent experiments (Padmavathi et al., 2005; Garodia et al., 2007; Widodo et al., 2007). Withanolide-D (witha-D) is an active element of WA that partially inhibits EMT acting on Wnt/β-catenin signaling and recovering E-Cadherin expression in a human pancreatic tumour cell line (Chaurasiya et al., 2008; Sarkar et al., 2014).

***Alpha-Solanine.*** This NPC presents pharmacological activities involving anti-proliferation, anti-apoptosis and anti-angiogenesis (Mohsenikia et al., 2013). Alpha-solanine also reduced ERK and PI3K/Akt phosphorylation. Likewise, this component also reduces the expression of MMP-2/9 and vimentin and induces the expression of E-cadherin (Shen et al., 2014).

### Potential Therapeutic Effects of Natural Plants Compounds

Currently, there is growing evidence for potential plant-derived compounds as inhibitors in several stages of tumourgenesis and inflammatory and fibrosis processes. In several clinical trials it has been demonstrated that NPCs have elicited anti‐aging, anti‐cancer and other health‐enhancing effects. A key target of the effects of NPCs may be in suppressing oxidative stress and the induction of 5′AMP-activated Kinase (AMPK), or suppression of the WNT/beta-catenin, PI3K/Akt/mTOR and RAS/MEK/ERK signaling pathways, among others, which results in cell death or prevents aging, diabetes, cardiovascular, cancer and other diseases (McCubrey et al., 2017).

One NPC is Berberine, which has been tested in a wide spectrum of clinical applications. Oral administration of berberine significantly reduced the familial adenomatous polyposis patients’ polyp size along with the inhibition of cyclin D1 expression in polyp samples. These statements suggest that berberine inhibits colon tumour formation through inhibition of Wnt/β-catenin signalling and might be a favorable drug for the prevention of colon cancer (Zhang et al., 2013; Farooqi et al., 2019). Additionally, it has been described that Berberine shows an extensive array of pharmacological effects, being effective against gastroenteritis, abdominal pain and diarrhea, and having antimicrobial, antidiabetic and antiinflammatory properties (Imanshahidi and Hosseinzadeh, 2008; Kulkarni and Dhir, 2010; Vuddanda et al., 2010). Another beneficial effect of berberine has been reported on the treatment of type II diabetes (Yin et al., 2008). This natural compound has an explicit potential as a drug in a wide spectrum of already defined clinical purposes (Tillhon et al., 2012). Numerous pharmacological reports have suggested the cardiovascular effects of berberine and *B. vulgaris*, such as preventing ischemia induced ventricular tachyarrhythmia, improving cardiac contractility and lowering peripheral vascular resistance and blood pressure (Marin-Neto et al., 1988).

Likewise, RSV is being examined in many clinical trials, on age-related disease, cancer, cardiovascular problems, chronic renal insufficiency and other disorders (Boocock et al., 2007; Brown et al., 2010; la Porte et al., 2010; Howells et al., 2011; Popat et al., 2013).

Clinical trials show that RSV has been shown to activate sirtuins and such activation is able to explain most of the beneficial properties of the mediterranean diet (MD). While observational studies and meta-analysis have demonstrated an antiageing effect of MD accompanied by a reduced risk of age-related pathologies, such as cardiovascular, metabolic and neurodegenerative diseases, as well as cancer (Russo et al., 2014; Gliemann et al., 2016).

Other studies that involved healthy volunteers established that RSV synchronized the carcinogen metabolizing enzyme cytochrome P450 and phase II detoxification enzymes, which repressed carcinogen metabolism and subsequently prevented carcinogenesis (Chow et al., 2010).

In the same way, Curcumin is being evaluated in numerous clinical trials for various disorders such as acute kidney injury, neurodegenerative diseases, cancer cardiovascular abnormalities, psychiatric disorders, osteoarthritis, type 2 diabetes mellitus, ulcerative colitis, rheumatoid arthritis, lupus nephritis, multiple sclerosis and other health problems (Allegra et al., 2017; White and Lee, 2019; Yang et al., 2019). Its efficacy appears to be related to the induction of glutathione S-transferase enzymes, inhibition of prostaglandin E2 (PGE2) production, or the suppression of oxidative DNA adduct formation. Oral curcumin was administered to patients with advanced colorectal cancer refractory to standard chemotherapies to explore its pharmacodynamics in humans (Sharma et al., 2004). In this study, the authors concluded that administration of 0.5 to 3.6 g/day for up to 4 months is associated with mild diarrhea as its only toxicity, and that a dosis of 3.6 g/day generates detectable levels of parent compound and conjugates in plasma and urine, causing inhibition of PGE2 production in blood leukocytes measured *ex vivo*. They proposed that an oral dose of 3.6 g/day is suitable for evaluation in Phase II trials (Sharma et al., 2004). In fact, curcumin has been found to be safe when administered at doses up to 10 g/day. All of these studies suggest that curcumin has enormous potential in the prevention and therapy of cancer (Aggarwal et al., 2003). Another study showed that curcumin is not toxic to humans up to 8 g/day when taken orally for 3 months (Cheng et al., 2001).

Likewise, epigallocatechin-3-gallate (EGCG) have been studied in a wide range of illnesses related to excessive oxidative stress, involving cancers, cardiovascular diseases, metabolic syndromes, diabetes, cerebral ischemic stroke, lung diseases, and neurodegenerative disorders (Chowdhury et al., 2016). Recently, EGCG has been studied for management and prevention of various kidney diseases, which are usually associated with oxidative stress and inflammation (Bao and Peng, 2016; Kanlaya and Thongboonkerd, 2019).

Meanwhile, baicalin decreases blood lipids and inflammation in patients with coronary artery disease and rheumatoid arthritis, supporting its further clinical application (Hang et al., 2018). This NPC exhibits high clinical value, having anti-inflammatory, anti-arrhythmic and anti-hypertensive effects (Huang et al., 2005; Huang et al., 2006; Dinda et al., 2017).

The therapeutic usefulness and anti-inflammatory properties of celastrol have been studied in numerous inflammatory diseases, involving rheumatoid arthitis, ankylosing spondylitis, systemic lupus erythematosus, inflammatory bowel disease, osteoarthritis, allergies, and skin inflammation (Cascão et al., 2017). Celastrol exhibits beneficial effects decreasing cardiovascular symptoms involving hypertension. Researchers investigated the treatment outcome against preeclampsia with a combined use of celastrol and nifedipine in clinical trials. A total of 626 patients with preeclampsia were enrolled, screened, and assigned randomly to groups receiving either nifedipine + placebo or nifedipine + celastrol orally. This study provides evidence for the potential role of celastrol serving as an effective and safe adjuvant to oral nifedipine against hypertension in patients with preeclampsia (Xiao et al., 2017). The therapeutic effects such as the anti-inflammatory, anticancer, and neuroprotective properties of celastrol can be mainly attributed to its capacity to inhibit NF-κB, a central player in inflammation, cancer and neurodegenerative diseases (Cascão et al., 2017).

Clinical investigation shows that gallic acid (GA) inhibits oxidative stress in diabetic patients. A small amount of GA prevents oxidative DNA injury and decreases markers which reflect inflammation and augmented risks of cancer and cardiovascular diseases (Ferk et al., 2018).

Clinical reports in asthma patients show that Genistein exerts antioxidant effects and could inhibit the pathway of NF-κB and TNF-α in these patients (Liu et al., 2010b).

It has been reported that Ginsenoside Rb1 (GS-Rb1) treatment was efficient in decreasing the extent of oxidative stress and inflammation in chronic kidney disease, whereas persistent deterioration was observed in the placebo group. Thus, extended treatments using GS-Rb1 may represent an interesting approach to slow the development of this disease at early stages (Xu et al., 2017).

### Limitations

Although NPCs are promising therapeutic agents, they need *in vivo* studies (animal models) mainly analyzing the specificity of their therapeutic action as well as toxic, mutagenic or side effects. Scientists must identify the components of each extract as well as the therapeutically active molecule/s. Moreover, previous prospective clinical trials are mandatory to recommend their use in clinical practice.

## Conclusion

EMT is a physiological and self-regulated process of tissue repair. However, pathologic EMT is characterized by its irreversibility and loss of self-regulation being a pathogenic part of many diseases. Thus, EMT is a therapeutic target with no established treatment yet. Natural products appear as therapeutic alternatives that need deep studies to be used in humans. Synergy and antagonism with other agents and interactions with prescription drugs should be studied in order to develop clinical trials.

The use of natural plant compounds versus standard drugs offers therapeutic advantages, such as the potential lower price and ease of being obtained, as they do not need to be artificially syntethized. Moreover, some of them are usually employed in the diet, like curcumin, although other routes of administration should be analyzed to calculate potential doses. Moreover, although many new drugs are made by synthetic chemistry and novel approaches to drug discovery such as combinatorial chemistry and computer-based design have been developed, they cannot replace the role of plant compounds in drug discovery, serving as chemical templates for the design and synthesis of new therapeutical drugs.

The relevance of this study lies on the necessity of finding effective therapies against EMT, which is a process involved in many diseases.

## Author Contributions

All the authors contributed to and approved the final manuscript.

## Conflict of Interest Statement

The authors declare that the research was conducted in the absence of any commercial or financial relationships that could be construed as a potential conflict of interest.
